# Development and Earliest Validation of a Portable Device for Quantification of Hallux Extension Strength (QuHalEx)

**DOI:** 10.3390/s23104654

**Published:** 2023-05-11

**Authors:** Elizabeth S. Hile, Mustafa Ghazi, Raghuveer Chandrashekhar, Josiah Rippetoe, Ashley Fox, Hongwu Wang

**Affiliations:** 1 Department of Rehabilitation Sciences, University of Oklahoma Health Sciences Center College of Allied Health, 1200 North Stonewall Ave., Oklahoma City, OK 73117, USA; josiah-rippetoe@ouhsc.edu (J.R.); ash-n-fox@ouhsc.edu (A.F.); 2 OU Health Stephenson Cancer Center, 800 NE 10th Street, Oklahoma City, OK 73104, USA; 3 Infant Neuromotor Control Laboratory, Division of Developmental-Behavioral Pediatrics, Children’s Hospital Los Angeles, Los Angeles, CA 90027, USA; mghazi@chla.usc.edu; 4 Department of Occupational Therapy, University of Florida, Gainesville, FL 32603, USA; rchandrashekhar@phhp.ufl.edu (R.C.); hongwu.wang@phhp.ufl.edu (H.W.)

**Keywords:** muscle strength, hallux, performance testing, load cell

## Abstract

Hallux strength is associated with sports performance and balance across the lifespan, and independently predicts falls in older adults. In rehabilitation, Medical Research Council (MRC) Manual Muscle Testing (MMT) is the clinical standard for hallux strength assessment, but subtle weakness and longitudinal changes in strength may go undetected. To address the need for research-grade yet clinically feasible options, we designed a new load cell device and testing protocol to Quantify Hallux Extension strength (QuHalEx). We aim to describe the device, protocol and initial validation. In benchtop testing, we used eight precision weights to apply known loads from 9.81 to 78.5 N. In healthy adults, we performed three maximal isometric tests for hallux extension and flexion on the right and left sides. We calculated the Intraclass Correlation Coefficient (ICC) with 95% confidence interval and descriptively compared our isometric force–time output to published parameters. QuHalEx benchtop absolute error ranged from 0.02 to 0.41 (mean 0.14) N. Benchtop and human intrasession output was repeatable (ICC 0.90–1.00, *p* < 0.001). Hallux strength in our sample (*n* = 38, age 33.5 ± 9.6 years, 53% female, 55% white) ranged from 23.1 to 82.0 N peak extension force and 32.0 to 142.4 N peak flexion, and differences of ~10 N (15%) between toes of the same MRC grade (5) suggest that QuHalEx is able to detect subtle weakness and interlimb asymmetries that are missed by MMT. Our results support ongoing QuHalEx validation and device refinement with a longer-term goal of widespread clinical and research application.

## 1. Introduction

Hallux strength is important across the lifespan in activities ranging from jumping [1] to balancing when standing [2,3,4]. In older adults, hallux plantarflexion strength independently predicts mobility performance and falls [5,6,7]. It follows that individuals with hallux plantarflexion weakness, from young competitive athletes to mobility-limited older adults, may benefit from hallux training [8]. Gauging the effectiveness and value of interventions in clinics and research requires valid metrics for the repeated quantification of strength.

Far less has been published about hallux dorsiflexion (extension) strength than plantarflexion (flexion), perhaps because there are fewer available options for quantifying extension strength [9]. Researchers have quantified flexion strength as the hallux or lesser toes push downward onto a force plate, sensor-impregnated platform, or while resisted by a plantar strap under tension [2,7,10,11,12,13]. Alternatively, flexion strength has been quantified as the hallux pulls back toward the heel while curled over a loop or bar [13,14,15,16]. Even a simple paper grip test of hallux flexion may inform the monitoring of neuropathy in persons with leprosy [17] or diabetes [18,19]. In contrast, there are few options for quantifying hallux extension strength [9]. We found only one published approach, wherein extension (albeit, of the lesser toes) was quantified by pulling upward against a dorsal strap under tension [12]. This lack of options may explain why a research team who measured toe flexor and extensor muscle sizes compared only the flexor data to strength [20].

Isokinetic machines are the gold standard for strength testing. They regulate the resistance applied to a joint while controlling the speed, range, and plane of any motion, and most allow isometric testing [21], but at high cost and with low portability. Hallux extension is not a standard isokinetic option, and even clinicians with isokinetic access also use Manual Muscle Testing (MMT), which is the clinical standard of care [22,23]. In MMT, a tester applies distal resistance in an attempt to overcome (break) or match (make test) the patient’s strength [24]. Simultaneously, the tester must stabilize the limb to isolate the desired motion and judge the patient’s force output on a subjective ordinal scale. The standard Medical Research Council (MRC) is a 6-point scale, from 0 (no contraction) to 5 (normal, able to withstand maximal pressure) [25]. The authors have discouraged the use of MMT in therapeutic trials due to its failure to reliably predict more quantitative strength values [26].

**Figure 1:**
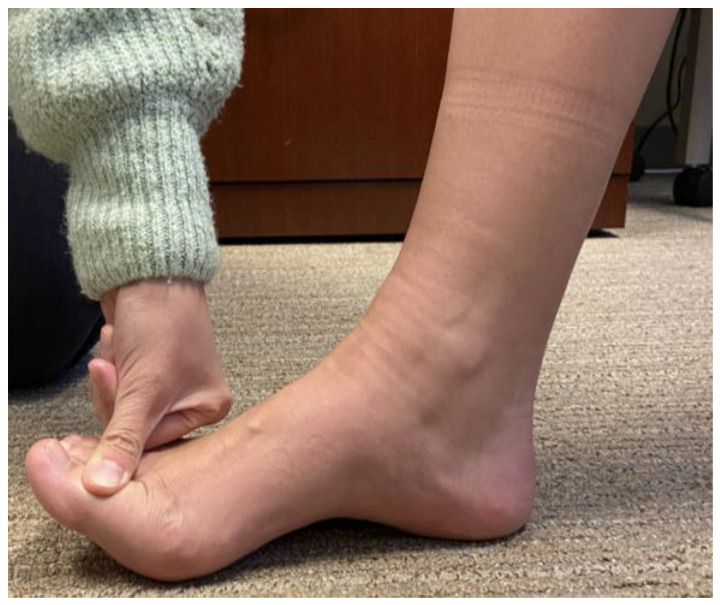
Manual Muscle Testing (MMT) of right hallux extension. Proximal stabilization provided during testing was omitted from the picture to visualize hallux extension at the metatarsophalangeal joint (MTP), the application of dorsal pressure near the interphalangeal joint, and the activated extrinsic muscle tendon (extensor hallucis longus).

To test hallux extension, the assessor applies force in the direction of flexion, either distal (if isolating extensor hallucis longus) or proximal to the interphalangeal joint, while the patient attempts to sustain the toe in extension (Figure 1) [27]. To a weaker assessor, a grade 4 toe may appear normal (grade 5). MMT is <75% sensitive to subtle but meaningful knee extensor weakness and asymmetry between limbs [28]. Failing to detect hallux asymmetry or weakness could delay the diagnosis of a neuromuscular condition, or an opportunity to improve balance or sports performance. Cadaver studies suggest that the MRC ordinal grade of 4 encompasses over 90% of the range of possible elbow flexion strength [22]. The authors attribute this phenomenon to low arm mass and lever distal to the elbow. The hallux has considerably smaller mass and lever; if 90% of possible hallux strength falls in grade 4, then manual testing will fail to recognize substantial functional improvement with intervention, or alternatively, substantial decline with pathology. Expanded scales utilize “plus” and “minus” [27,29], but those qualifiers are subjective [22].

Hand-held dynamometers (HHD) can provide more objective and precise strength quantification than MMT [30]. Compared to the 0–5 ordinal ratings, the continuous scale of digital HHD achieves greater discrimination of strength scores [30], but the tester must still match the patient’s force while stabilizing the dynamometer against the tested limb. Furthermore, by definition, a muscle of MRC grade 3 or below is not able to sustain the position of the limb against resistance [27], so HHD is not applicable [22,30]. Finally, the reliability and validity of HHD are lower for distal muscle groups [23,31], and strategies that improve validity for some muscles [32,33] may be more challenging to implement for hallux extension.

Ultimately, clinicians and researchers lack a low-cost and portable option that is sufficiently valid for comparing the full range of hallux extension strength between people, detecting subtle weakness or strength asymmetries, and monitoring for change over time in response to intervention or disease. In response to the psychometric limitations of MMT and HHD, we developed a “hands-off” device and protocol for the Quantification of Hallux Extension (QuHalEx) strength. QuHalEx eliminates the application of a manual or mechanical downward force on the extended hallux and reports results on a continuous scale to eliminate subjective ratings. Our portable device reflects a collaboration between clinical rehabilitation researchers and bioengineers with a goal of multisite utility in a variety of settings and for a variety of purposes. We aim to introduce the QuHalEx device and protocol, establish benchtop accuracy, and test the intrasession reliability of hallux extension and flexion strength testing in healthy young and mid-life adults.

## 2. Materials and Methods

### Device Conceptualization: Design Requirements

The lead clinician inventor (ESH), a board-certified specialist in neurologic physical therapy, consulted the lead bioengineering inventor (HW) for a device to quantify isometric hallux extension strength on a continuous scale and without applying downward pressure or adding weight or tension to restrict the patient’s initial toe motion. Instead, a fixed restraint would contact the dorsal hallux near the interphalangeal joint to limit continued upward (extension) motion while a sensor quantifies isometric hallux strength. The sensor should activate in response to low levels of volitional force, and in a low range of metatarsophalangeal joint extension motion, in order to quantify output from toes with severe weakness or joint immobility. In addition, the device design should:

1. Allow testing of either the right or left foot.
2. Accommodate adult feet as large as United States Men’s size 14 and variations in toe size (length, width) and shape.
3. Restrain the foot proximal to the metatarsophalangeal (MTP) joint to isolate hallux extension and discourage ankle dorsiflexion, a secondary action of the extrinsic hallux extensor muscle, extensor hallucis longus [34].
4. Include markings (e.g., ruler-like) or another mechanism to guide the tester in reproducing a patient’s foot position in subsequent serial assessments.
5. Sample the output for at least 5 s, and at a frequency sufficient to capture intra- and inter-individual variability in initial and sustained voluntary muscle recruitment. (Most extremity muscles have a recruitment frequency of about 10–11 Hz [35])
6. Record force output over time and present key results (peak force) to the tester immediately so they may confirm the collection of a valid trial.

The device should also quantify the force generated when a patient flexes the MTP joint to press the hallux downward, and should include a visual feedback mechanism; evidence suggests that visual feedback improves peak output and other performance metrics during testing of isometric strength and eccentric torque [36,37,38,39].

## 3. Device Prototype

In response to these requests, the bioengineering team fabricated QuHalEx, a portable device to quantify hallux strength, especially hallux extension, in an iterative and collaborative process with ongoing feedback from the clinical research team. QuHalEx measures force using a load cell sensor and transmits the signal to a Dell XPS13 computer over USB port to be recorded for later analysis. Technical specifications are provided in Table 1 and detailed in the text that follows.

### 3.1. Hardware

**Figure 2:**
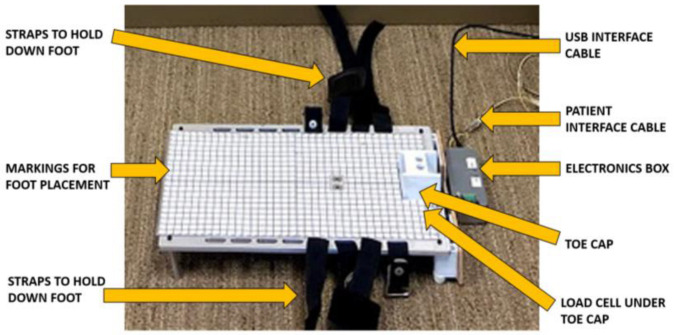
Hardware details for the QuHalEx device.

The QuHalEx base is an aluminum plate raised off the ground by four 51 cm “legs” (Figure 2). A laminated grid serves as a positioning reference for future assessments of the same patient. A 48 × 48 × 34 mm ABS plastic toe cap produced on a Fused Deposition Modeling (FDM) 3-D printer is bolted to the front end of the load cell. The rear edge of the load cell is then bolted to the front of the base plate so that forces generated at the toe cap are transmitted through the load cell.

The toe cap is centered on the base to allow testing of a right or left foot, and is open proximally to accommodate hallux insertion (Figure 2). Two screws attach the cap to the base, allowing easy substitution with a cap of alternative size, though our primary cap has fit every hallux tested. We beveled the cap’s proximal edges for comfort because they rest in the 1st toe webspace during the test. The cap’s distal opening enhances comfort but also standardizes the location of dorsal resistance during isometric hallux extension. We trialed a dome-shaped closed cap, but in some cases toenail contact with the inner roof created discomfort or passive interphalangeal joint (IPJ) flexion; IPJ flexion caused the toe to retract from the cap with continued MTP extension. The earliest prototype (by ESH and a student team, see acknowledgements) used a single Velcro strap to restrict extension by securing the toe down to the base; however, it could be loosened or even opened by strong toes. With the current open square design, we have observed no passive IPJ flexion with extension strength testing of any adult hallux (unisex shoe size 5.5 to 15).

Once the hallux is inside the cap, the tester secures the foot to the base with a series of four hook and loop cinch straps with Velcro closure (Figure 2). Adjacent straps are oriented in opposite directions to facilitate a neutral foot position. When oriented in a single direction, tightening the straps could pull the foot into passive pronation or supination.

In addition to hallux extension strength, QuHalEx also quantifies flexion strength. The QuHalEx load cell is activated when the hallux pushes downward onto the rigid platform in the same motion as most force plate testing protocols [7,13]. Seated hallux strength correlates only moderately to standing strength measured by force platform, perhaps because the latter includes activity from other postural muscles especially ankle plantarflexors [13]. To isolate hallux flexion, we employ a seated QuHalEx protocol.

### 3.2. Electronics

**Figure 3:**
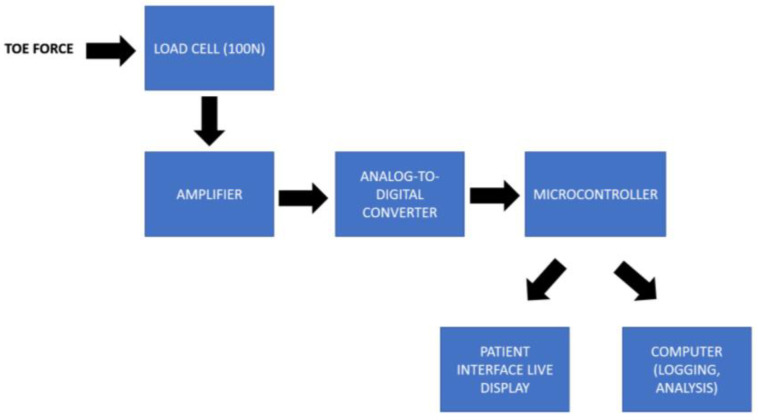
Overview of the QuHalEx device electronics.

The QuHalEx device uses a one-axis load cell to measure force generated by the hallux inside the toe cap. The force measured is positive when the hallux pulls isometrically upward against the cap’s roof (MTP extension), and negative when the hallux pushes isometrically downward (MTP flexion). In each direction, the load cell detects up to 100 N of force (equivalent to ~10 kg mass). We chose 100 N to avoid a ceiling effect based on the highest extension force we recorded during developmental testing (60 N from a young adult male runner). The load cell excitation voltage is set at 5 V, and voltage output is amplified (Figure 3) using an instrumentation amplifier (AD620, Analog Devices, Wilmington, NC, USA) with a 2.048 V offset (LM4040, Texas Instruments, Dallas, USA). The resulting amplified voltage is then measured with a 12-bit analog-to-digital converter module (ADS1015, Adafruit, New York City, NY, USA). The analog-to-digital converter also measures the load cell excitation voltage and transmits voltage data to a microcontroller (Arduino Pro Mini, 16 MHz, 5 V, Sparkfun Electronics, Boulder, CO, USA) for conversion to force based on a calibration curve. Any fluctuations in power supply (excitation voltage) are corrected based on the excitation voltage measured. Force output from the load cell with 50 Hz sampling rate is carried by USB serial port to a laptop computer. Simultaneously, a microcontroller transmits force output in real-time to activate one or more display LEDs in a patient feedback interface. Patient interface electronics include a microcontroller (Arduino Pro Mini, 16 MHz, 5 V, Sparkfun Electronics, Boulder, CO, USA) and nine display LEDs. Refer to Figure 3 and Section 3.4 for further description.

The device has two physical buttons on the electronics box (Figure 2). During test administration, the operator presses one button to command the device to perform a tare operation, and the other to measure force for a pre-selected duration, currently 10 s.

### 3.3. Software

TeraTerm version 4.105 (Tera Term Project, Tokyo, Japan) is a text-based serial terminal that receives the force data over USB and displays and logs the data as a function of time as it is transmitted from the device. TeraTerm is not used to control the device; a Graphical User Interface is under development for that purpose.

### 3.4. Patient Interface

**Figure 4:**
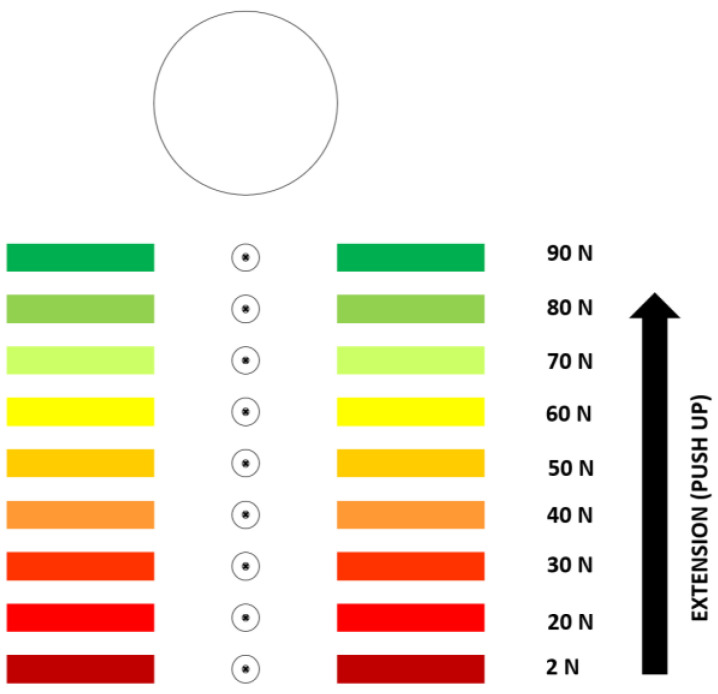
Patient interface (visual feedback system). The activation thresholds for each light are presented for illustration only. They are not provided on the actual interface.

Our patient interface is a visual display with a size of 508 × 406 mm (L × W), with nine LEDs arranged in a vertical row 30.5 cm long (Figure 4). The LEDs activate in sequence, and in proportion to the force detected by the load cell. After the second LED (20 N) is activated, activating each subsequent LED requires an additional 10 N of force. The first LED to be activated requires a force of only 1.57 (2 N). This threshold was selected to reward force in the desired direction (flexion or extension) that surpasses physiologic noise. We want ≥1 LED to light even when weak toes (Medical Research Council grade 2) are extended to contact the cap’s roof. The light provides immediate feedback to patient and tester that the patient’s toe is extending as directed. Our best estimate of the threshold between noise and weakest volitional hallux extension is 1.57 N. To estimate this threshold, we measured load cell output while lab members held their own hallux as still as possible inside the cap (noise estimate), and then again with the smallest hallux extension force they could generate. We aim to measure maximal strength and within-trial fatigue, so the interface doubles as incentive to sustain best effort for the test duration. We set the activation threshold for the highest LED at 90 N to exceed the highest peak force (~60 N) we observed in healthy adult lab members with a strength corresponding to MRC grade 5.

## 4. Data Collection for QuHalEx Device Validation

### 4.1. Benchtop Calibration

For bench-top testing, we used precision weights to apply eight known loads of 9.81 N through 78.5 N, at 9.81 N intervals. These loads were based on known masses of 1.00 kg through 8.00 kg, with 9.81 m/s^2^ acceleration due to gravity. To apply each load, we used a pivoted beam structure we designed to mimic toe extension forces. We generated an upward force from the end of the beam positioned inside the toe cap by suspending precision weights from the opposite end. We performed accuracy testing in sets of 250 repetitions at each load, removing the load to tare the device after each rep. In total, we applied three sets for each load, in the order of lowest to highest load. For benchtop intrasession reliability, we simulated human repeated testing by applying each load (9.81 N, 19.6 N, 29.4 N, 39.2 N, 49.1 N, 58.9 N, 68.7 N, and 78.5 N) as one set of three repetitions.

### 4.2. Human QuHalEx Testing

We obtained Institutional Review Board approval to test healthy adults at least 20 years of age who ambulate independently with no assistive device and deny chronic pain or a history of lower limb or axial surgery or major body injury (e.g., fracture). Participants were excluded for evidence of imbalance or movement dysfunction.

The lead physical therapist (ESH) trained a single non-clinician assessor (AF) to perform Manual Muscle Testing (MMT) to introduce the desired motion while the toe was fully visible, before insertion in the QuHalEx cap. We selected break testing to measure maximal force generation [40]. Participants extended their hallux as far as possible toward the ceiling. The assessor compared the active range of MTP extension to the available passive motion. If grossly equivalent (Medical Research Council grade 3 [41]), the assessor applied downward pressure at the interphalangeal joint as illustrated in Figure 1 with instructions to “hold your toe up as strong as you can. Don’t let me push you down”.

**Figure 5:**
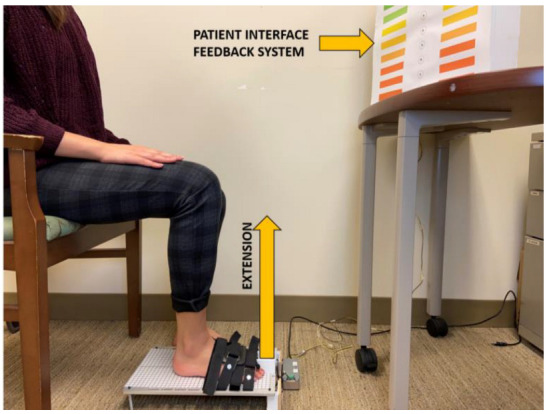
Physical setup of the QuHalEx device with patient interface display.

For QuHalEx testing (Figure 5), we place the device on a level floor and instruct the seated subject to fully insert one hallux into the toe cap until their web space contacts the proximal edge. Because pain may interfere with muscle performance [42] and some lab members reported mild to moderate discomfort with web space or distal hallux contact against the toe cap, we applied a commercially available silicone gel-impregnated toe sleeve prior to testing. The toe sleeve also promotes hygiene within the cap.

The untested foot rests on the floor next to the device and the participant faces the LED interface. The tester repositions the subject or device to achieve neutral hip rotation, 90 degrees of knee flexion, and neutral ankle position before strapping the foot on the metal plate. The 90–90–90 seated position (Figure 5) is common [15], and neutral or slightly dorsiflexed ankle positioning facilitates maximum isometric toe flexion [43]. We instructed participants to sit upright with their low back in a lordotic posture and away from the backrest, and to not lean forward or to either side.

Without instruction for the four smaller toes during isometric hallux extension testing, we observed one of three natural strategies: (1) extension upward with the hallux; (2) flexion downward toward the platform, often with ankle inversion; or (3) minimal motion of the smaller toes. Because different strategies changed the hallux force reading, our standardized instruction became, “pull all of your toes up toward the ceiling”. After we confirm correct technique in a practice trial, we repeat, “remember to pull the big toe up as hard as you can”. For hallux flexion, we instruct, “push the big toe down on the platform as hard as you can without lifting the heel”. Maintaining heel contact discourages the ankle plantarflexion role of the extrinsic flexor, hallucis longus [44]. After a loud “go” command [37], we repeat the phrase “pull, pull, pull, keep going, keep going…” for the duration of the hallux extension trial (alt: “push” for hallux flexion). Studies support the use of verbal encouragement, especially combined with visual feedback, to improve amplitude and timing of muscle output [37,38,45,46]. For visual feedback, we direct participants to watch the LED interface, try to activate the top light, and continue giving their strongest effort until they are told to stop. To encourage maximal effort, we do not inform patients that they are unlikely to reach the threshold to activate the top light. We use a familiarization trial [23] at submaximal effort to confirm activation of at least one light before three maximal isometric trials with 15–20 s rest between each [47]. After testing, we clean the device with commercial wipes impregnated with isopropyl alcohol and ammonium chloride.

## 5. Data Processing and Analysis for Device Validation

### 5.1. Benchtop

For accuracy testing, we computed the mean of 250 measurements for each of three trials at a given load. For intra-session reliability, we conducted a two-way mixed effects interclass correlation coefficient (ICC) with absolute agreement across three trials of each of the eight different loads.

### 5.2. Humans

We used descriptive statistics (mean, standard deviation, median, interquartile range, minimum, maximum) to summarize raw QuHalEx extension and flexion output. For intra-session reliability, we conducted two-way mixed effects ICC with absolute agreement across three trials. We computed ICCs with 95% confidence intervals, by side (right–left) and test (extension–flexion). For all analyses, we used IBM SPSS 28 and a significance level of 0.05.

To generate load–time curves, we applied a second-order 24 Hz low-pass Butterworth filter and defined the time of force onset (Time Zero) for each trial as the point when force reached the 1.57 N threshold to activate the lowest LED on the patient interface. After aligning all trials at Time Zero, we visualized 5 s of data collection because some adults may not reach peak force until 5 s after force onset [47,48]. For each trial, we defined 100% peak as the maximum force output within the 5 s window, and 90% peak as 0.9× (100% peak). For each plot, we indicate the time of each event relative to Time Zero, for example the time (latency) to 90% and 100% peak. We plotted hallux extension output for three trials from a single toe, and separately we plotted three trials from three different toes, all rated MRC grade 5 (strongest) by a single MMT assessor.

## 6. Results

### 6.1. Benchtop Validation

#### 6.1.1. Accuracy

**Figure 6:**
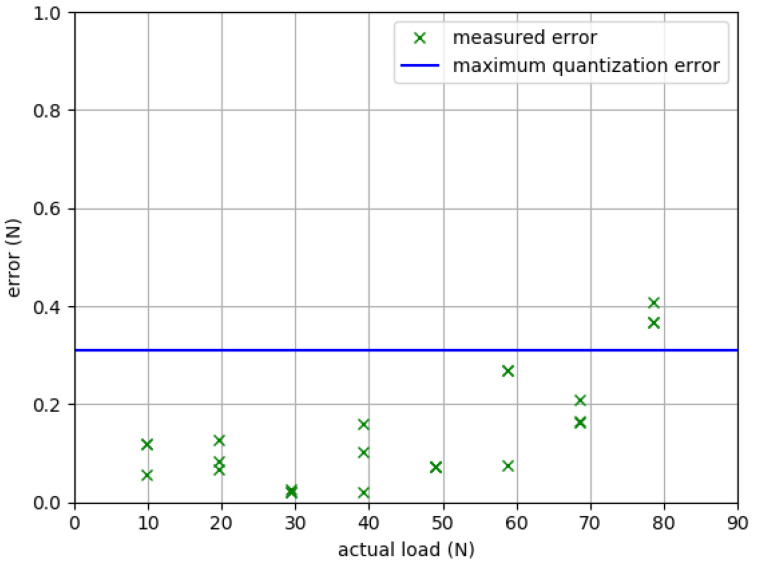
Benchtop testing results: device accuracy as absolute measurement error. Each “×” marks the mean of 250 tests for each of three trials at each of eight loads (9.81, 19.6, 29.4, 39.2, 49.1, 58.9, 68.7, and 78.5 N). The horizontal line for benchmarking is the 0.31 N theoretical maximum quantization error for the system’s analog-to-digital converter.

Refer to Table 2 and Figure 6 for calibration results. Absolute error ranged from 0.02 N to 0.41 N (mean 0.14 N), and remained within the maximum possible analog-to-digital converter quantization error (~0.31 N for our system) until at least 68.7 N.

#### 6.1.2. Intrasession Test–Retest Reliability

The ICC for benchtop repeated trials within a single session was 1.00 (*p* < 0.001).

### 6.2. Human Validation

A total of 38 participants completed QuHalEx reliability testing. The demographic variables for the cohort are summarized in Table 3.

#### 6.2.1. Intrasession Reliability and Construct Validity

**Figure 7:**
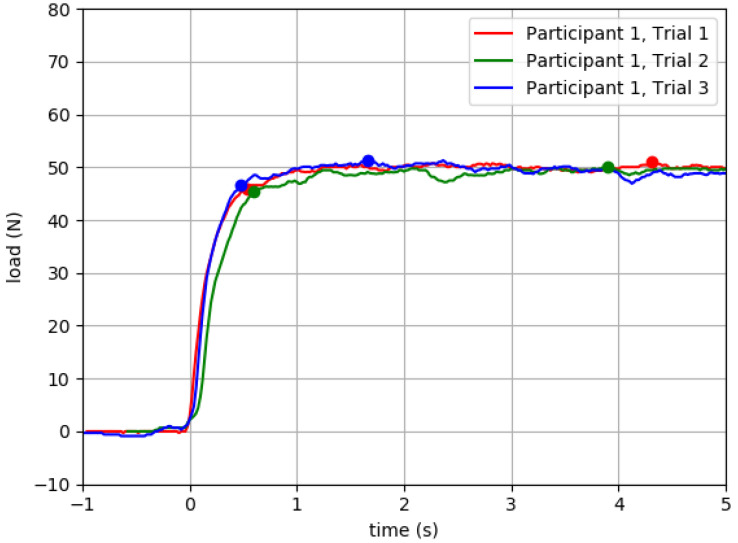
QuHalEx isometric extension load–time curves for three trials from one participant’s right hallux. Ninety percent peak force (1st dot) occurred 0.48–0.62 s after force onset; 100% peak (2nd dot) came 1.21–3.81 s later. Peak force was 51.0, 50.0, and 51.3 N for trials 1–3, respectively. After 90% peak, the load remained within 8.58% of peak. For illustration, we include 1 s of data with toe in cap before Time Zero. For each trial, 90% peak force is derived from the value of the maximum load registered.

In Table 4 we present raw QuHalEx peak force output and intrasession ICC values, organized by side (right or left) for isometric flexion and extension. All ICCs were statistically significant (*p* < 0.001). In Figure 7, we present representative load–time plots of isometric hallux extension output for repeated trials from a single toe.

#### 6.2.2. Potential to Mitigate Ceiling Effects of Manual Muscle Testing

**Figure 8:**
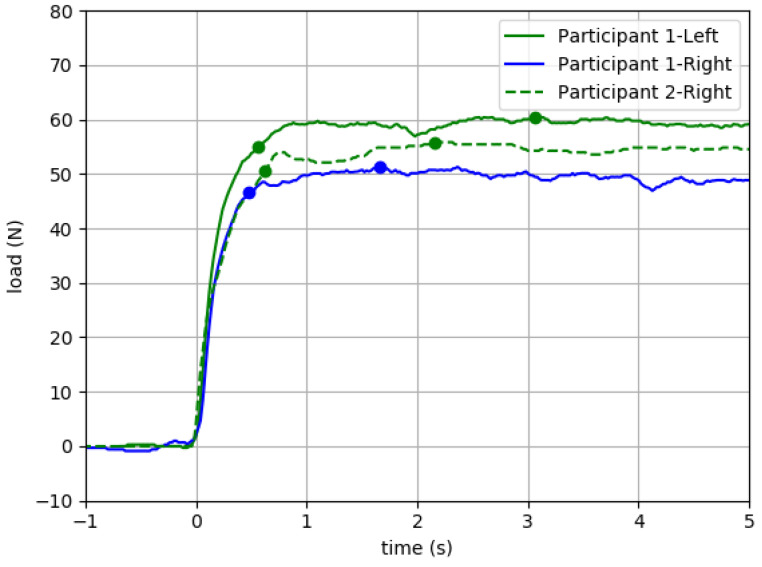
QuHalEx isometric extension load–time plots for three different toes with Medical Research Council grade 5 strength. Solid blue = trial of highest peak force (51.3 N) for Participant 1’s right hallux; solid green = trial of lowest peak force (60.9 N) for Participant 1’s left hallux. Dashed green = representative trial from a different participant’s MRC grade 5 hallux.

Of three QuHalEx trials from Participant 1’s right hallux (Figure 7), the highest peak extension force (51.3 N) was recorded in trial 3. When this same right hallux trial (solid blue) was plotted in Figure 8 alongside the participant’s trial of lowest peak force (60.9 N) for the left hallux (solid green), the load–time curves are visually similar but exhibit a 9.6 N interlimb peak force asymmetry even though both were rated MRC grade 5 on manual testing. A load–time curve from the MRC grade 5 hallux of a different participant (dashed green) is similar in shape, and after 90% peak force, the value of the sustained load falls between those of Participant 1’s right (weaker) and left (stronger) toes.

## 7. Discussion

We developed a load cell device to quantify hallux extension strength (QuHalEx), and a protocol that is acceptable to healthy adults and clinicians yet feasible for administration by assessors with no healthcare training. In our benchtop and human testing, QuHalEx peak force output appears to be accurate and repeatable within a single testing session. Visual analysis of load–time curves from healthy adult toes provides evidence of construct validity and suggests that QuHalEx may discriminate subtle differences in strength that currently go undetected when using the Medical Research Council (MRC) Manual Muscle Testing (MMT), which is the clinical standard of care. Included among these subtle differences are within-person strength asymmetries of potential significance to physical performance and health.

### 7.1. Initial Validation

QuHalEx force output was accurate and repeatable when we calibrated against known loads in benchtop testing (Table 2). For eight loads from 9.81 N to 78.5 N, mean absolute error was 0.14 N (≤1.4% of the load). ICCs for intrasession repeated testing were 1.00 at the bench and 0.905–0.916 [95% CI: 0.842–0.952] for right and left hallux flexion and extension in our sample of adult participants. All ICCs were statistically significant (*p* < 0.001), and our human ICC values are in the range of 0.77 to 0.95 published by teams using other approaches to quantify hallux flexion strength [11,13,16].

In our clinical testing with 38 healthy young and mid-life adults, QuHalEx isometric peak force ranged from 23.1 to 82.0 N (mean 52.0 ± 12.3 N) for hallux extension and 32.0 to 142.4 N (mean 88.9 ± 29.8 N) for hallux flexion. In comparison, Chatzistergos and colleagues reported hallux strength of 24–128 N (mean 66 N) for extension and 21–209 N (mean 101 N) for flexion in an older cohort (mean 58 years) with diabetes [49]. The upper limits of Chatzistergos’ ranges substantially exceed our QuHalEx ranges, but they were measured with HHD, a very different device and protocol. Additionally, the limit of our load cell is ±100 N because of our initial focus on the measurement of hallux extension which is typically weaker than flexion. In contrast, for hallux flexion, our average QuHalEx peak force exceeds means published from pressure-mat testing in healthy young adults: 62.6 N ± 43.9 N [13] or 31.5 ± 16.8 N (women)–65.8 ± 28.0 N (men) [11]. In our cohort, mean and median QuHalEx peak were higher for the right hallux when compared to the left, and over 90% of our participants report right leg dominance for kicking a ball. We found evidence of this trend (stronger right hallux) in the literature [13,49].

The QuHalEx isometric extension load–time curves (Figure 7 and Figure 8) are visually similar across trials, resemble those published by Yamauchi and Koyama for isometric hallux flexion [43], and exhibit the rise to peak that is characteristic of maximum isometric force development [50]. In three repeated trials of a single toe (Figure 7), the latency to 90% peak extension force consistently occurred ~0.5 s after force onset (intertrial range = 0.14 s). Within a trial, after reaching 90% peak, the force output sustained within 9% for the 5 s volitional hold. Across repeated trials, the value for 100% peak remained within 2% (1.0 N) of the median; repeated testing is recommended for trials exceeding 5% [51]. In the same three QuHalEx extension trials, the latency to 100% peak varied by 2.6 s and occurred nearly 4.5 s after force onset. This is consistent with published rationale for collecting 5 s of isometric strength data [47,48]. The magnitude of variability we observed within—and between—QuHalEx trials is reasonable when compared to published dynamometry and electromyography output from lower extremity muscle groups that include the hallux flexors [13,51].

Because clinicians use patterns of lower limb weakness (e.g., proximal more than distal, bilaterally symmetrical) to make diagnostic decisions and referrals, the optimal strength screening tool is sensitive to subtle differences and free of ceiling effects. Our QuHalEx results (Figure 8) suggest that hallux extension force can vary by at least 15% among two toes that both perform at the MRC grade 5 MMT ceiling. We found this difference as a right–left strength asymmetry for a single participant, and we suggest that the stronger hallux could lose at least 9 N of strength before MMT would detect decline and a potential need for referral. Even if linked functional deficits led this patient to rehab, with grade 5 strength, toe exercises would not be prioritized in the plan of care. The MRC artificial ceiling is quantified for ankle extension [23] but we may be the first to quantify the issue in hallux extension.

In addition to the strongest toes, we intend for QuHalEx to discriminate hallux strength in the weakest grades (MRC grade 1–3). This is relevant to the care of patients with distal weakness from neuromuscular pathologies; hallux strength monitoring could capture disease progression, or a response to pharmacologic or rehabilitative intervention. Just as MacAvoy and colleagues demonstrated for elbow flexion [22], we suspect that MRC grade 4 encompasses the majority of available hallux extension strength. We have found it clinically challenging to distinguish hallux strength in the MRC grades of 2 and 3, and some patients cannot readily transfer to the recommended grade 2 “gravity reduced” test position. In response, we attempted to quantify hallux extension strength by hand-held dynamometry (HHD), but we quickly grew concerned about reliability when testing weak toes. Consistent with the MRC definition, grade 3 toes yielded to our attempts to match force, sometimes before the HHD could register output. For this reason, QuHalEx does not require a tester to match a patient’s force output, and the device provides passive rather than active resistance to motion.

### 7.2. Limitations

Our design has limitations. QuHalEx measures global hallux strength in either extension or flexion. It does not isolate testing to a single muscle, nor does it distinguish intrinsic and extrinsic contributors to the force output, but this is consistent with our focus on functional hallux output for activities and participation. To isolate the output to the desired hallux flexion or extension activity, we tare the device only after the foot and hallux are positioned on the platform. Even so, a change in seated weight bearing or active ankle plantarflexion will confound the hallux reading. To minimize this occurrence, we carefully position and instruct the patient, and then closely monitor their limb throughout the test; we are working to automate this procedure.

We anticipate difficulty using QuHalEx to quantify extension output from the weakest toes (MRC grade 1) because the current design requires the hallux to lift upward from the platform ~20 degrees before contacting the roof of the toe cap to activate the load cell. We will continue to refine the design to mitigate this floor effect. In an early prototype that measured only hallux extension, a dorsal Velcro strap secured the hallux to the platform and also activated the load cell. Although the load cell measured force with minimal toe lift, the output was not sufficiently reliable to move to human testing.

In benchtop calibration, we identified error as high as 0.42 N with the 78.5 N known weight. This error exceeds the 0.31 N maximum analog to digital converter quantization error for our system. Quantization error is caused by the truncation of the continuous analog voltage output as it is digitized by the analog-to-digital converter. Our elevated measurement error at loads approximating 80 N could reflect noise in the electronics of the system, or errors in the load cell calibration curve which would become more pronounced at higher loads. Only 5% of our healthy young adult cohort approached 80 N in extension force; however, 80 N is near the average of peak force for hallux flexion. We are prioritizing this issue in ongoing refinement and upgrading our load cell limits.

### 7.3. Future Directions

In this earliest study, we calibrated against known weights, but we have no gold standard for benchmarking the human trials. While MMT is the clinical standard of care, it is not a gold standard such as electromyography or isokinetic strength testing, neither of which were available to our team. We recognize the need to perform further QuHalEx validation with a larger and more diverse sample, and with expansion to intersession test–retest reliability, concurrent validity, and known-groups validity. We also plan to collect normative data for both sexes across a broader age range.

We seek a more reliable, objective, and sensitive alternative to MMT that is still feasible in all settings. To inform clinical decision making in real time, we are refining a QuHalEx graphical user interface (GUI) to provide the assessor with immediate display of robust processed data while allowing tablet or mobile control. We are also digitizing the real-time visual feedback for a more portable patient interface. Ultimately, we envision in-home self-assessment with integrated training functions. Handgrip is used to monitor muscle function in aging and disease and is predictive of adverse outcomes [52], but new evidence suggests that toe grip declines even earlier [53]. This raises novel implications for our device in remote health monitoring. Critically, ours may be the only device of its kind capable of measuring both hallux flexion and extension to generate a hallux strength index; implications of lower extremity agonist–antagonist neuromuscular balance include joint stability for injury prevention [54].

## 8. Conclusions

Hallux strength is linked to physical performance and may be an underprioritized target for evaluation, monitoring, and intervention of individuals across the lifespan, and for reasons that span neurologic, endocrine, orthopedic, sports, and more. We described the design and protocol for our new approach to the Quantification of Hallux Extension strength (QuHalEx) and presented initial evidence that our approach is feasible, accurate, and reliable for collecting load–time output from seated isometric hallux strength testing, and with greater discrimination than the current standard clinical approach.

## 9. Patents

The QuHalEx strength testing device and testing protocol described in this manuscript are patented by The University of Oklahoma Health Sciences Center as U.S. patent 11402284, Apparatus and Method for Measuring Toe Flexion and Extension Strength. E.S.H, M.G., and H.W. are tri-inventors on the patent.

## Figures and Tables

**Table 1:** Technical specifications of the QuHalEx device.

Feature	Value
Dimensions L × W × H (mm)	419 × 229 × 89
Weight (kg)	1.80
Maximum Load (N)	±100
Resolution (N)	0.31
Accuracy (N)	0.02–0.42

**Table 2:** QuHalEx calibration results: absolute error (mean of 250 reps) by trial (T).

Actual Load (N)	|Error T1| (N)	|Error T2| (N)	|Error T3| (N)
9.81	0.118	0.118	0.0561
19.6	0.0671	0.127	0.0825
29.4	0.0217	0.0258	0.0206
39.2	0.159	0.103	0.0207
49.1	0.0716	0.0729	0.072
58.9	0.0747	0.268	0.269
68.7	0.208	0.165	0.162
78.5	0.367	0.407	0.366

**Table 3:** Participant characteristics (*n* = 38).

Variable	Unit or Category	Mean (SD) Min–Max	Frequency Count (%)
Age	Years	33.5 (9.6) 20–54	
Height	Inches	66.0 (6.8) 32–75	
Weight	Pounds	159.1 (34.3) 94–249	
Sex	Female Male		20 (52.6) 18 (47.4)
Ethnicity	Hispanic or Latinx Not Hispanic or Latinx		3 (7.9) 35 (92.1)
Race	Asian Black or African American More than One Race White Not Specified		11 (28.9) 2 (5.3) 2 (5.3) 21 (55.3) 2 (5.3)
Shoe Size	United States Unisex	8.9 (1.9) 5.5–15	
Kicking Foot *	Right Left Not Specified		35 (92.1) 2 (5.3) 1 (2.6)

* The preferred foot for kicking a ball as reported by the participant.

**Table 4:** QuHalEx intrasession test–retest reliability in adult participants.

Hallux Test	Side	Peak Force		
		Mean (SD) Min–Max	Median [IQR]	ICC [95% CI] *
Extension (*n* = 38)	Right	52.8 (12.6)	52.9	0.907
		29.6–82.0	[19.9]	[0.848–0.947]
	Left	51.3 (12.0)	51.7	0.916
		23.1–80.3	[17.4]	[0.862–0.952]
Flexion (*n* = 37)	Right	90.1 (28.9)	89.4	0.905
		39.0–140.6	[46.2]	[0.842–0.946]
	Left	88.0 (30.9)	86.8	0.910
		32.0–142.4	[41.2]	[0.851–0.950]

* All *p* < 0.001 Key: CI = Confidence Interval; ICC = Intraclass Correlation Coefficient; IQR = Interquartile Range; N = Newtons; SD = Standard Deviation.

## Data Availability

The data presented in this study may be requested from the authors. For deidentified human participant data, contact the corresponding author (E.S.H). For benchtop testing data contact the last author (H.W.). Institutional approvals and data use agreements may be required. The deidentified data are not yet publicly available because the study is ongoing.
